# ‘Concept creep’ in perceptions of mental illness — an experimental examination of prevalence-induced concept change

**DOI:** 10.1007/s00406-023-01737-0

**Published:** 2024-01-17

**Authors:** Sven Speerforck, Vanessa Jürgensen, Mirjam Göbel, Nicholas Meyer, Georg Schomerus

**Affiliations:** 1 Department of Psychiatry and Psychotherapy, Medical Faculty, University of Leipzig, Semmelweißstraße 10, 04103 Leipzig, Germany; 2 Institute of Psychiatry, Psychology and Neuroscience, King’s College London, London, UK

**Keywords:** Stigma, Concept creep, Mental illness, Medicalization, Diagnoses expansion, Prevalence

## Abstract

The perception of what constitutes mental illness is influenced by various social and medical developments. Prevalence-induced concept change is a phenomenon where decreasing the prevalence of a category leads people to expand their judgment of that concept. In this study, we tested whether changing the prevalence of statements describing mental illness results in a change in the concept of mental illness. Based on a population survey (*n* = 1031), we created a validated set of 273 brief statements depicting either clear symptoms of mental illness, clear examples of healthy behaviour, or ambiguous situations. We presented a subset of statements to 138 students, asking them to judge whether each statement represented mental illness, or not. After 96 statements, we reduced the prevalence of clearly mentally ill statements in one group, while the proportion of statements denoting clear mental illness remained the same in the other group. In the group where the proportion of clearly mentally ill statements was reduced during the experiment, a concept change of mental illness evolved: participants were more likely to identify a statement as denoting a mental illness. The results indicate that the perceived prevalence of symptoms of mental illness is important for conceptualizing mental illness and that decreasing prevalence broadens the concept of mental illness. These findings add a novel perspective to current debates around diagnostic thresholds, the treatment-prevalence paradox, the medicalization of emotions, and the focus of anti-stigma campaigns.

**Supplementary Information:**

The online version contains supplementary material available at 10.1007/s00406-023-01737-0.

## Introduction

How people perceive a particular concept depends not only on its content, but also its prevalence. This was demonstrated by Levari et al. [1], who showed that for both simple concepts such as whether the colour of sequentially presented dots was blue or not, to more complex judgements such as the dangerousness of computer-generated human faces or the ethical justifiability of a research study, *decreasing the prevalence of a category* led to participants *expanding their judgment of that concept*. For example, as the prevalence of blue dots, threatening faces, or unethical research proposals were reduced, participants were more likely to rate non-blue dots as blue, non-threatening faces as threatening, or ethically ambiguous research proposals as unethical. Colloquially termed ‘concept creep’ [2], this influential finding suggests that rather than remaining stable, our perception of a concept changes according to its prevalence.

The perception of mental illness among the general public has shifted over time. For example, over a 10-year period, the concept of depression was increasingly judged as being familiar, comprehensible, and on lying on a continuum between mental illness and mental health, whereas, the opposite was found for schizophrenia [3]. In parallel, discussion in the media has increasingly moved toward framing mental disorder through the lens of mental health and mental wellbeing [4], while symptoms of severe and enduring mental illness, such as mania, hallucinations and delusions, have received proportionally less emphasis [5]. While it may be that concepts of mental illness are independent from how frequently they are reported, the phenomenon of prevalence-induced concept change may apply in this context, and offer a particularly relevant model for exploring this relationship.

Understanding how concepts of mental illness are determined is important for several reasons. On the one hand, for those experiencing mental distress, conceptualizing their symptoms as being part of a mental illness may be valuable, as it validates their experiences, and situates them within the domain of a medical condition, thereby allowing individuals to take the next step to seek support [6]. On the other hand, the extent to which mental distress is viewed as an illness is relevant to debates surrounding the medicalization of everyday emotions [7], where feelings that were previously considered to be within the normal range of human experience are viewed as disordered, and treated, for example, with medication or psychotherapy. In turn, this has consequences on service use, and availability of resources for treating individuals experiencing more severe mental symptoms. Similarly, the label ‘mentally ill’ has been linked to the process of stigmatization, by which individuals are more likely to experience discrimination [8]. Conflating the concept of ‘mental illness’ with ‘mental wellbeing’ may therefore have the positive consequence of reducing stigma, and promoting recovery and social integration. For people living with chronic, distressing and disabling difficulties such as psychosis, however, the blurring of boundaries between these concepts may have the unintended consequence of minimising the severity of their experiences.

Changes in the prevalence of symptoms of severe mental illness, therefore, could lead to changes in illness recognition, service use, and stigma. There have been few theoretical attempts to examine the relationship between the prevalence of symptoms of mental disorder, and the extent to which these symptoms are conceptualized by the general public as being part of a mental illness. Applying the principle of prevalence-induced concept shift of Levari et al. [1], we investigate in this exploratory study whether prevalence-induced concept change is relevant to the construct of mental illness.

## Methods

### Selection of statements

To capture the concept of “mental illness”, an online quota-sample of the general population (USUMA Market and social research, Berlin) rated short statements about different conditions and behaviors of an unspecified person, on a 7-point Likert scale, indicating to what extent each statement represents mental illness (*n* = 1031). The distribution within the net sample was adjusted to the population living in Germany by weighting age, gender, household size and the regional distribution of the federal states to data from the population living in Germany, using iterative proportional fitting from the “Mikrozensus 2018” [9].Statements from the mentally ill category were developed along the 5th chapter (mental and behavioral disorders) of the ICD-10-GM (e.g., “A person cannot distinguish whether things are real or happening in his or her mind.”). We excluded eating disorders (F50), sexual dysfunction (F52), postpartum mental or behavioral disorders (F53), intelligence disorders (F70–F79), developmental disorders (F80-F89), and disorders affecting children and adolescents from our statements. We also developed statements from the ambiguous category along the ICD-10-GM, but in an attenuated way (e.g., “A person has few social contacts and tends to withdraw”). Statements from the category mentally healthy were freely chosen (e.g., “A person smacks while eating”).

Of these statements, we then selected 240 statements with the lowest standard deviation (SE) based on Levari et al. (study 7) [1]. According to their respective mean rating in the general population, the 240 statements were categorized into the three categories “mentally ill” (e.g., “A person cannot distinguish whether things are real or happening in his or her mind.”), “ambiguous” (e.g., “A person has few social contacts and tends to withdraw”), and “mentally healthy” (e.g., “A person smacks while eating”). A detailed description of this process can be found in the supplement. The translated list of the 273 statements together with their mean and standard deviation is available within the Open Science Framework (osf.io/w7muh/).

### Experiment

We then used these statements in the experiment, which took place between October and December 2021. 150 students participated; students studying medicine or psychology were excluded in order to avoid professional bias. All participants were recruited using notices in university buildings and libraries or via digital distribution channels, provided written informed consent and received an incentive of 20 euros.

Participants categorized each of the 240 statements as either part of a mental illness or not, using a yes/no answer format. We divided participants systematically into two conditions: half of the participants were assigned to the *stable* condition (*n* = 71). In this condition, statements of all three categories (mentally healthy, ambiguous, mentally ill) occurred with equal probability across all 240 trials. With reference to Levari et al. [1], we refer to the probability of selecting a statement from the mentally ill category as the *signal prevalence*. Accordingly, in the stable condition, the signal prevalence was 33.3%. The other half of the participants (*n* = 67) belonged to the *decreasing* condition. In this condition, the signal prevalence decreased steadily from the 4th set onward. That is, the signal prevalence was 33.3% on the trials 1–96; 25% on trials 97–120; 16% on trials 121–144; 8.3% on trials 145–168, and 4.12% in trials 169–240. Thus, toward the end, participants in the decreasing condition saw statements almost exclusively that belonged to the mentally healthy and ambiguous category according to the objective rating of mental illness.

After the participants had completed the experimental part of the study, the online survey tool RedCap was used to collect the socio-demographic data of the participants.

### Statistical analysis

We analysed the data using a generalized linear mixed model using the R statistical software platform, version 4.0.2 (R Foundation for Statistical Computing). The binary measurement of whether a statement was judged as “mentally ill” or “mentally healthy” served as the dependent variable. The two conditions (stable and decreasing conditions), served as independent between-participant variable. The trial number (1–240) and the objective norming measurement from the pre-study from “definitely not” (1) to “definitely” (7) served as independent within-participant variables. We used the Akaike information criterion (AIC) for choosing the best-fit for the data. We captured conditional R^2^ for model performance. We calculated Odds ratios for further interpretation using sjPlot [10]. Post hoc power analysis for the hypothesized interaction effect between trial number and condition was performed using R package simr version 1.0.7 with 1000 simulations [11]. The experiment constructed with OpenSesame [12], our data set, and the corresponding R script are available within the Open Science Framework (osf.io/w7muh/).

## Results

**Fig. 1:**
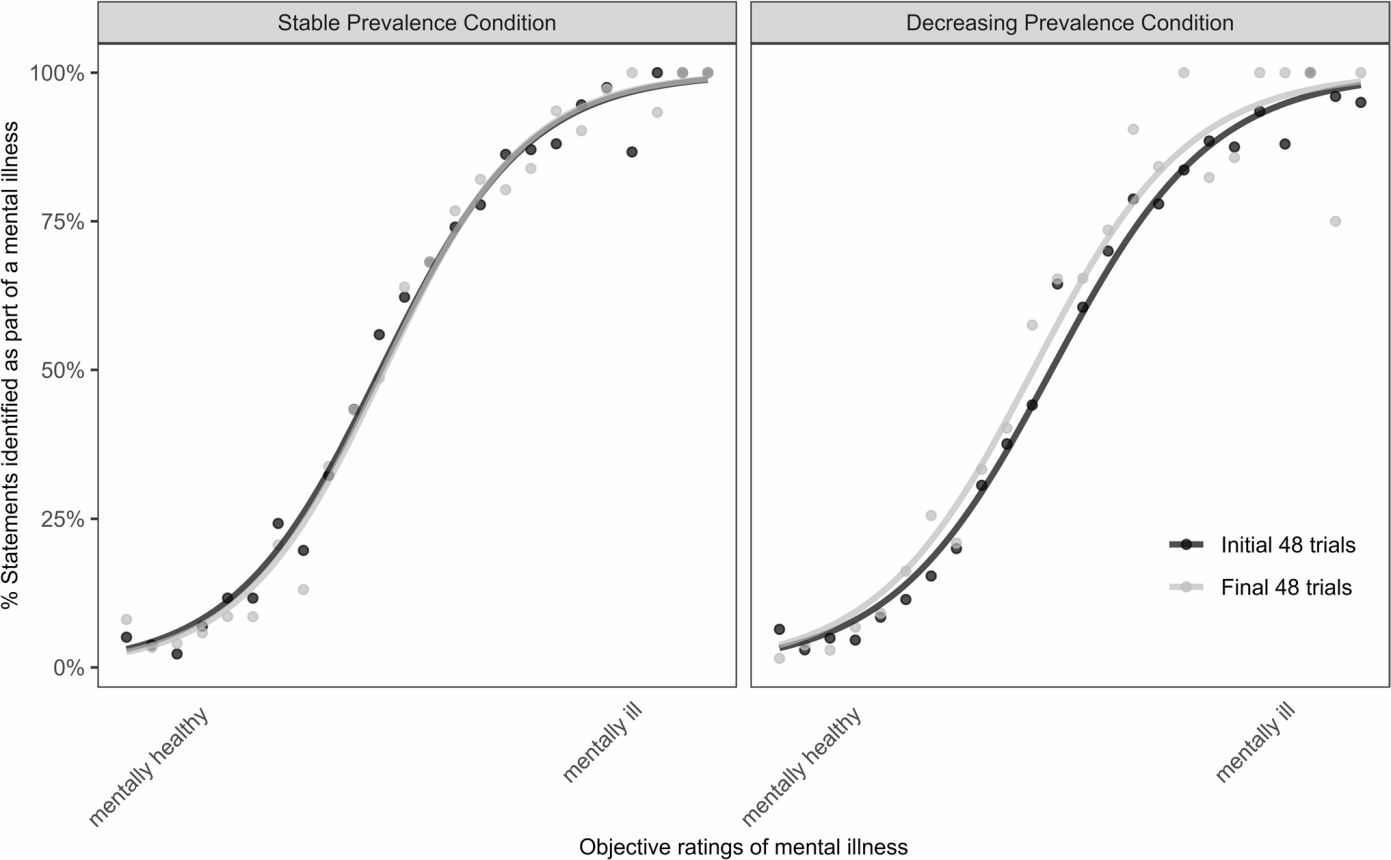
Experimental results. The left panel shows the stable prevalence condition, and the panel on the right shows the decreasing prevalence condition. The x-axes show the norming measurements from the pre-study (objective ratings of mental illness) and the y-axes show the percentage of trials in which participants identified that statement as part of a mental illness. The black line shows the evaluation for the first 48 trials and the grey line shows the evaluation for the last 48 trials. In the stable condition, both lines overlap and no change can be detected. In the decreasing prevalence condition, a shift toward concept expansion evolved: a reduced prevalence of “clearly mentally ill” statements resulted in a higher likelihood of rating particularly “ambiguous” and “clearly mentally ill” statements as indicating mental illness

We excluded ten participants due to failed attention checks (two questions about previously given content), and two participants who could not finish the experiment due to technical issues. Our final sample thus consisted of 138 students (30 male, 106 female, 2 gender unspecified, M_age_ = 23.49 years, SD = 3.53 years). Supplementary Table 1 shows gender, age, and study subject for participants. The best-fit model used as fixed factors the condition, trial number and objective norming measurements plus including the interaction of trial and condition and trial and objective norming measurements. As random factors, we included intercepts for participants and the slopes for trial number. Model fit was significantly improved by both. The generalized linear mixed model showed that the condition*trial interaction predicted participants’ responses, *b* = 0.51, *SE* = 0.22, *z* = 2.33, *R*^*2*^_*GLMM(c)*_ = 0.67. Odds ratio was 1.66. Monte Carlo simulations yielded a power of 65.40% (62.36, 68.35) for this particular interaction term. Table 1 comprises the regression coefficients of the generalized linear mixed model. The respective Odds ratios can be found in Supplementary Table 2. Figure 1 shows the percentage of statements that participants identified as signifying mental illness, separated by prevalence condition illustrating a shift toward concept expansion.

**Table 1:** Regression coefficients of generalized linear mixed model fit by maximum likelihood

Fixed effects	Estimate	SE	*z*	*p*
Intercept	– 7.1479	0.2006	– 35.627	< 2e-16 ***
Prevalence condition	– 0.3301	0.1624	– 2.033	0.0421 *
Trial	– 2.1412	0.3452	v6.203	5.52e-10 ***
Norm mean	12.1787	0.2796	43.550	< 2e-16 ***
Prevalence condition * trial	0.5054	0.2168	2.331	0.0197 *
Trial* norm_mean	3.5335	0.5271	6.704	2.03e-11 ***

All test statistics represent those reported by the glmer() function; Signif. codes: 0 ‘***’ 0.001 ‘**’ 0.01 ‘*’ 0.05 ‘.’ 0.1 ‘

## Discussion

Concepts of mental illness have changed over time, under the influence of scientific developments, together with secular shifts in social, political and cultural factors. Here, in line with previous work [1], we demonstrate that judgements of what constitutes mental illness is also a function of its prevalence. Although effect sizes are small, our results suggest that as identifiable symptoms of mental illness become less prevalent, participants’ concepts of mental illness expand to include symptoms that they had previously excluded in the higher-prevalence condition.

A fall in the prevalence of symptoms of severe mental illness, for example through improved prevention and treatment of mental illness, or a reduction in the public awareness of such symptoms through, for example, a shift in media concepts toward mental wellbeing and mental health, may result in the broadening of the perception of what constitutes mental illness. A greater number of less severe symptoms become integrated into the concept of mental illness. This mechanism may contribute to how concepts of mental illness are formed and change within the general population, with several important implications.

An expanding concept of mental illness may increase help-seeking behaviors and demands on mental health services from people who would have not previously sought help. This mechanism may also offer an additional explanation to the treatment-prevalence paradox in depression [13, 14], where more effective and widespread treatment of depression has not been accompanied by a commensurate decrease in its prevalence. It could also be a mechanism by which ‘ordinary human suffering’, and experiences that were previously considered subclinical, are medicalized [15].

The findings are also of relevance to stigma and discrimination in mental illness. On the one hand, a broader concept of mental illness could be accompanied by more empathy and tolerance toward people with mental illness, thus contributing to destigmatization. If less disturbing or troublesome symptoms are categorized as part of the concept of mental illness, people with mental illness might generally be seen as less different and frightening. In the UK, a wider concept of mental illness has been accompanied by less stigma toward “people with a mental health problem” [16]. Conversely, the same development could be detrimental to people with severe and treatment-resistant mental illness. An expansion of the concept of mental illness toward the healthier pole might increase perceived differentness, fear, and stigma toward those with apparent symptoms of severe mental illness, reinforcing stigma for those most affected [3].

Our current findings bring attention to the importance of using precise and considered language in Psychiatry, and the importance of clinicians and researchers recognizing how changes in language, definitions, and categorizations might influence how diagnostic concepts are perceived. We speculate that promoting awareness and discussion in society of a wide range of mental symptoms, particularly including more severe symptoms associated with mania, psychosis or addiction, may reduce the hypothesized concept creep in perceptions of mental illness.

Our study is of course experimental, and generalizations remain speculative. Replication in larger, diverse samples, testing the robustness of the effect in real-world samples is necessary, as statistical power for the interaction effect was just high enough to ensure statistical significance (*p* = 0.02). Should the finding be replicated, it remains unclear whether concept creep can, or should be, changed at all. Future research should carefully consider the desirable and undesirable consequences associated with the phenomenon, and whether the former outweighs the latter. An important future study would ask whether the converse direction of effect — increasing prevalence of symptoms leading to a narrower concept of mental illness — is observed, and if a change of concept is accompanied by a corresponding change in attitudes. It is important to consider that our study focused on a non-specialist/general population, sampled at one point in time. Therefore, we don`t know whether the observed effect will differ in clinicians, patients or family and friends of people with symptoms of mental illness or if it is influenced by longitudinal exposure over longer timescales. All participants in our experiment were students. Consequently, our analyses refer to a younger, well-educated population. Moreover, we examined the overarching concept of “mental illness” and cannot discern the extent to which prevalence change is relevant to any specific mental illness.

Nonetheless, prevalence-induced concept change in the context of mental illness might adds a novel perspective to current debates around the treatment-prevention paradox, the medicalization of emotions, and the focus of anti-stigma campaigns.

## Supplementary Information

Below is the link to the electronic supplementary material.

Supplementary file1 (PDF 301 KB)

Supplementary file2 (DOCX 50 KB)

## Data Availability

The experiment constructed with OpenSesame [12], our data set, and the corresponding R script are available within the Open Science Framework (osf.io/w7muh/).
