# Supplementary material for: ‘Concept creep’ in perceptions of mental illness — an experimental examination of prevalence-induced concept change
Source: Eur Arch Psychiatry Clin Neurosci. 2024 Jan 17;276(1):33–7. doi: 10.1007/s00406-023-01737-0 (PMC12904865; doi:10.1007/s00406-023-01737-0)
Supplement: Supplementary file 1 — Supplementary file1 (PDF 301 KB) [file 406_2023_1737_MOESM1_ESM.pdf]

mentally healthy

item	statement	a priori classification	mean	sd
vset8_1_a16	A person who likes to go to the cinema by themselves	mentally healthy	1,606382979	1,01830647
vset2_2_a10	A person likes to do a lot of sports	mentally healthy	1,705263158	1,156606226
vset3_2_a9	A person who smokes at parties	mentally healthy	1,81443299	1,269267316
vset7_1_a12	A person is on a trip of several months and misses their family	mentally healthy	1,680851064	1,271801414
vset6_1_a13	A person does not like nuts	mentally healthy	1,627659574	1,278305688
vset5_2_a15	A person has to do some work, but is persuaded by a friend to spend an evening together in the bar.	mentally healthy	1,92	1,284562575
vset6_2_a16	A person has an earworm for days	mentally healthy	1,946808511	1,289886394
vset8_2_a18	A person takes the bigger piece of the cake	mentally healthy	1,978494624	1,310314388
vset5_2_a13	A person who forgets to turn down the heater after opening the window	mentally healthy	1,98	1,325583538
vset2_1_a13	A person who likes to do risky sports	mentally healthy	1,895833333	1,333607428
vset3_2_a11	A person who finds it very easy to meet new people	mentally healthy	2,06185567	1,367894009
vset2_2_a12	A person forgets their wallet at home	mentally healthy	1,978947368	1,368171224
vset2_2_a15	A person who likes to sleep at noon	mentally healthy	1,715789474	1,37348104
vset9_2_a11	A person smacks while eating	mentally healthy	1,97029703	1,374448584
vset1_2_a15	A person forgets a date with their friend	mentally healthy	2,253731343	1,382135488
vset2_2_a13	A person who loves their job and therefore works more than necessary	mentally healthy	2,115789474	1,382744403
vset5_1_a13	A person who likes to drive fast	mentally healthy	2,19	1,390407251
vset4_2_a17	A person gets scared watching horror movies	mentally healthy	2,053763441	1,393818585
vset8_1_a12	A person who burps in public	mentally healthy	1,968085106	1,394706796
vset8_1_a17	A person has a morning routine that they usually follow because it gives them a good start to the day	mentally healthy	2,106382979	1,394829817
vset8_2_a12	A person runs through the traffic light on red	mentally healthy	2,127659574	1,400722107
vset2_1_a15	A person who always gets up early	mentally healthy	1,802083333	1,418819314
vset9_1_a12	A person who puts thoughts together before an argument to be quick-witted	mentally healthy	2,264705882	1,420685029
vset3_2_a10	A person who enjoys being the center of attention	mentally healthy	2,494845361	1,422466398

vset2_2_a16	A person who eats very mentally healthy	mentally healthy	1,778947368	1,437928085
vset8_1_a15	A person oversleeps an important appointment	mentally healthy	2,010638298	1,44053963
vset4_2_a12	A person lies when asked how he or she likes the food	mentally healthy	2,215053763	1,451007614
vset8_2_a5	A person constantly tells inappropriate jokes or stories	ambiguous	2,691489362	1,451929349
vset5_1_a17	A person is so engrossed in a book that they forget the world around them	mentally healthy	1,95	1,459036627
vset2_1_a16	A person who cries at movies	mentally healthy	2	1,465390194
vset3_1_a18	A person puts themselves in the shoes of others and tries to understand their point of view	mentally healthy	2,285714286	1,485323388
vset5_2_a17	A person who keeps changing hobbies	mentally healthy	2,34	1,485417331
vset3_2_a14	A person who stands up for their opinion, even if it is unwillingly heard	mentally healthy	2,298969072	1,48720199
vset2_2_a14	A person is enthusiastic about new things	mentally healthy	1,810526316	1,48964323
vset9_2_a15	A person who questions things they read in the news	mentally healthy	2,138613861	1,490165783
vset3_1_a8	A person has an overcorrect way of speaking	ambiguous	2,775510204	1,495909283
vset4_2_a14	A person is a big fan of an actress and watches every one of her movies	mentally healthy	2,075268817	1,498089792
vset6_2_a15	A person must discipline themselves to do their paperwork	mentally healthy	2,670212766	1,49830225
vset8_1_a18	A person is looking forward to their vacation because they have been reluctant to do their job lately	mentally healthy	2,117021277	1,508801297
vset4_2_a3	A person does not take into account social norms, but unrestrainedly expresses his or her opinion	ambiguous	2,782608696	1,510593268
vset5_1_a18	A person who goes jogging when he or she is stressed	mentally healthy	1,91	1,511538116
vset10_1_a15	A person who installs smoke detectors in their home because they are afraid of fire	mentally healthy	2,065420561	1,512657464
vset6_2_a12	A person watches an episode of a series even though it is late and they have to get up early the next day.	mentally healthy	1,968085106	1,513041

vset10_1_a11	A person feels neglected because his or her friends do not contact him or her.	mentally healthy	2,710280374	1,51725513
vset3_2_a16	A person has a queasy feeling when flying	mentally healthy	2,618556701	1,523724186
vset3_2_a12	A person who is reserved in unfamiliar contexts	mentally healthy	2,587628866	1,525837047
vset10_1_a12	A person tries the lottery in the hope of becoming a millionaire	mentally healthy	2,186915888	1,530389278
vset9_1_a15	A person who has no interest in moving away from their place of birth	mentally healthy	2,059405941	1,535068612
vset10_1_a14	A person gets goosebumps when he or she hears a touching song	mentally healthy	2,102803738	1,535335864
vset3_1_a15	A person chuckles quietly to themselves in public because they have thought of something amusing	mentally healthy	2,632653061	1,535744254
vset6_2_a14	A person is impatient when waiting in a queue	mentally healthy	2,276595745	1,540871526
vset2_2_a19	A person who occasionally feels the desire to have a cigarette	ambiguous	2,094736842	1,544197679
vset3_2_a15	A person likes to collect unusual things, such as stuffed animals.	mentally healthy	2,567010309	1,54722343
vset1_1_a16	A person who is frustrated when he or she cannot put a plan into action	mentally healthy	2,777227723	1,553322947
vset1_2_a13	A person who is jealous of his/her partner	mentally healthy	2,89	1,555634919
vset3_2_a13	A person who does not like to travel alone, but prefers to travel in groups	mentally healthy	2,391752577	1,558288999
vset3_1_a14	A person approaches new tasks enthusiastically	mentally healthy	2,448979592	1,560613473
vset6_2_a11	A person who expresses their character and creativity through their clothes	mentally healthy	2,14893617	1,565470466
vset9_2_a16	A person is nervous before a class meeting	mentally healthy	2,485148515	1,565974849
vset4_1_a14	A person is messy	mentally healthy	2,215053763	1,566285153
vset5_2_a16	A person who likes to listen to loud music even though it is late and it might disturb the neighbors.	mentally healthy	2,52	1,566569952
vset4_1_a11	A person blasphemes about others	mentally healthy	2,376344086	1,566732813
vset7_2_a13	A person who is curious and interested in their fellow human beings	mentally healthy	2,053191489	1,568281172

vset8_2_a16	A person does not go to a bar alone because he or she does not feel comfortable there without a friend.	mentally healthy	2,553191489	1,569994324
vset2_1_a14	A person sings to themselves in public	mentally healthy	2,416666667	1,573910094
vset5_2_a19	A person thinks that he or she is very popular with others	mentally healthy	2,87	1,574127945
vset6_2_a6	A person has a pronounced urge to talk, even in an environment that is unfamiliar to him or her	ambiguous	2,957446809	1,578858262
vset8_1_a14	One person was so drunk the night before that they can't remember parts of it	mentally healthy	2,180851064	1,579184259
vset9_1_a3	A person who takes different natural remedies every day	ambiguous	2,803921569	1,579818361
vset10_2_a16	A person waffles on whether he or she has made the right decision	mentally healthy	2,869158879	1,584620139
vset5_1_a14	A person who has difficulty learning things by heart	mentally healthy	2,56	1,584776057
vset4_2_a10	A person believes in God	mentally healthy	1,978494624	1,601483651
vset6_1_a18	A person tends to be skeptical towards strangers	mentally healthy	2,861702128	1,603766158
vset5_2_a14	A person who gets a laughing fit and can hardly calm down	mentally healthy	2,47	1,604633443
vset6_1_a14	A person is fastidious and for this reason does not like to lend personal belongings	mentally healthy	2,85106383	1,606153803
vset8_2_a17	One person drinks coffee every day	mentally healthy	1,691489362	1,606616666
vset4_1_a15	A person blushes when something makes them uncomfortable	mentally healthy	2,258064516	1,607747721
vset10_2_a13	A person insults someone in an argument	mentally healthy	2,551401869	1,609134567
vset1_2_a11	A person who has a positive attitude towards life	mentally healthy	1,895522388	1,610599221
vset9_1_a14	A person who likes themselves	mentally healthy	2,411764706	1,612776593
vset9_1_a10	A person lives very goal-oriented and therefore tries to assert him or herself against others	mentally healthy	2,578431373	1,613288104
vset1_1_a17	A person who has an unpleasant dream	mentally healthy	2,376237624	1,616774537
vset10_1_a10	One person is too lazy to cook and prefers to order food	mentally healthy	2,112149533	1,621361545
vset2_2_a9	A person searches a little longer for a word	mentally healthy	2,915789474	1,622076145
vset6_1_a4	A person has no close friends	mentally ill	2,79787234	1,623614627

vset1_2_a16	A person who is very ambitious in their profession	mentally healthy	2,243781095	1,629501038
vset1_1_a14	A person who is envious of other people's successes	mentally healthy	2,638613861	1,633935535
vset9_2_a10	A person quits their job even though they don't have a new one yet, which means they have to register as unemployed	mentally healthy	2,594059406	1,638158831
vset7_2_a7	A person dresses in a strange or unkempt manner	ambiguous	2,882978723	1,645171384
vset9_1_a13	A person who delays a visit to the dentist.	mentally healthy	2,666666667	1,649082153
vset7_2_a15	A person has a strong urge to exercise and therefore does sports several times a week	mentally healthy	2,425531915	1,649303285
vset4_2_a13	One person spends 20 minutes a day on social media	mentally healthy	2,010752688	1,651709415
vset10_1_a7	A person finds it difficult to refrain from smoking in places where smoking is prohibited	mentally ill	2,841121495	1,654892875
vset8_2_a14	A person embellishes a story to make it sound more exciting	mentally healthy	2,914893617	1,656914962
vset5_2_a12	One person can eat a lot	mentally healthy	2,81	1,661780717
vset7_2_a8	A person feels like they can literally pull out trees	ambiguous	2,457446809	1,663426271
vset10_1_a3	A person who is very sensitive to criticism	ambiguous	2,952830189	1,664086052
vset7_1_a14	A person who is late for meetings with friends	mentally healthy	2,212765957	1,664766705
vset2_1_a9	A person seems quirky and eccentric	ambiguous	2,958333333	1,666491219
vset7_2_a12	A person who is not interested in politics and news	mentally healthy	2,085106383	1,669843689
vset9_2_a12	A person who is upset when someone forgets their birthday	mentally healthy	2,404040404	1,671606779
vset2_2_a11	A person enjoys having time to themselves and their hobbies	mentally healthy	1,904255319	1,672478998
vset1_1_a11	A person who is upset after an argument with a person he or she is close to	mentally healthy	2,584158416	1,673720358
vset11_1_a11	A person neglects their household because they have a lot of stress at work at times	mentally healthy	2,916408669	1,674501623
vset6_2_a13	A person likes to walk through the forest watching animals	mentally healthy	1,893617021	1,675041871
vset4_2_a11	A person is concerned that everyone at a gathering gets along well and has a good evening	mentally healthy	2,397849462	1,675525777

vset4_1_a18	A person is certain that he or she is not up to a task, but approaches it anyway to test his or her limits	mentally healthy	2,752688172	1,678870699
vset6_1_a12	A person is confrontational and also stands up for others	mentally healthy	2,301075269	1,686164584
vset1_1_a12	A person who has many ideas	mentally healthy	2,169154229	1,688562638
vset5_1_a1	A person wakes up early in the morning on their own for months without needing to or wanting to	ambiguous	2,888888889	1,689637619
vset7_2_a9	A person has an excessive fondness for fantasy	ambiguous	2,968085106	1,694089203
vset5_1_a16	A person who is afraid alone in the forest at night	mentally healthy	2,64	1,696818175
vset4_2_a16	A person is unhappily in love but cannot stop thinking about the person	mentally healthy	2,612903226	1,700589783

ambiguous

item	statement	ICD-10-GM	a priori classification	mean	sd
vset3_1_a3	A person feels a great confidentiality even in unfamiliar contexts, with strangers and tells personal things	F30-F39	ambiguous	3,275510204	1,405296404
vset3_2_a5	A person disregards social obligations	F60-F69	ambiguous	3,536082474	1,45101872
vset3_1_a4	A person has unusual difficulty concentrating during a conversation	F00-F09	ambiguous	3,867346939	1,454731031
vset3_1_a10	A person who finds it difficult to meet new people		mentally healthy	3,540816327	1,472555583
vset3_1_a6	A person has a tendency to be argumentative and conflictual	F60-F69	ambiguous	4,030612245	1,481529452
vset3_2_a6	A person tends to have intense but volatile relationships	F60-F69	ambiguous	3,144329897	1,492967478
vset8_2_a13	A person cannot admit that they were wrong		mentally healthy	3,010638298	1,520432033
vset5_1_a9	A person is convinced of being socially awkward	F60-F69	ambiguous	3,47	1,546942243
vset6_2_a3	A person appears indifferent and jaded in social encounters	F00-F09	mentally ill	3,85106383	1,551672333

vset11_1_a13	A person laughs when he or she is insecure		mentally healthy	3,022657055	1,566235735
vset1_1_a13	A person who sometimes has difficulty concentrating		mentally healthy	3,311881188	1,569851983
vset1_2_a1	A person has a persistent craving for external stimuli and influences	F60-F69	ambiguous	3,706467662	1,571116788
vset7_2_a17	A person feels overwhelmed by new tasks		mentally healthy	3,127659574	1,574214551
vset8_2_a11	A person often feels that he or she is misunderstood, treated unfairly, or held overly accountable	F60-F69	ambiguous	3,670212766	1,575267841
vset3_1_a2	A person keeps forgetting important events after an unexpected bereavement in his or her close environment	F40-F48	mentally ill	3,816326531	1,588274943
vset5_1_a10	A person often misspeaks due to nervousness and stutters in conversations with strangers.	F40-F49	ambiguous	3,71	1,597314666
vset9_2_a5	A person neglects their duties at work	F00-F09	ambiguous	3,36	1,598736875
vset7_2_a4	A person cannot listen attentively for more than five minutes	F10-F19	mentally ill	3,691489362	1,599909915
vset5_2_a6	A person shows a mood inappropriately elevated to the situation	F30-F39	ambiguous	3,89	1,601104417
vset10_1_a1	A person has a persistent longing for affection and acceptance in any social context	F60-F69	ambiguous	3,626168224	1,604964965
vset10_1_a4	A person reacts very slowly to external stimuli such as sounds or touch.	F00-F09	mentally ill	3,504672897	1,609956239
vset9_1_a1	A person who is already sure in the morning that the day will not go well	F30-F39	ambiguous	3,735294118	1,610156321
vset3_2_a4	A person who speaks very slurred and indistinctly	F10-F19	ambiguous	3,536082474	1,620582815
vset8_2_a10	A person has a tendency to defer to the wishes of elders and others	F60-F69	ambiguous	3,234042553	1,622592721

vset5_1_a8	A person tends to dramatize and express feelings theatrically	F60-F69	ambiguous	3,83	1,627137289
vset1_2_a12	A person who sometimes has difficulty concentrating		mentally healthy	3,109452736	1,630325182
vset8_2_a9	A person has a very vague or complicated way of speaking	F20-F29	ambiguous	3,127659574	1,634533524
vset9_1_a11	A person struggles for words when nervous or upset		mentally healthy	3,128712871	1,635012944
vset9_2_a18	A person ponders whether they have done something wrong after a friend does not reply to a message for an unusually long time	F60-F69	ambiguous	3,079207921	1,635134051
vset2_1_a7	A person basically feels passive resistance to suggestions and performance demands from other people	F60-F69	ambiguous	3,40625	1,638857914
vset6_1_a9	A person does not trust the diagnoses of his or her doctors.	F40-F48	ambiguous	3,531914894	1,650689837
vset4_2_a4	A person tends to overestimate themselves and their abilities	F30-F39	ambiguous	3,139784946	1,652346144
vset4_1_a4	A person has difficulty classifying sounds correctly	F10-F19	ambiguous	3,150537634	1,66123472
vset11_1_a2	A person who is overcome by severe fatigue after minor activities	F30-F39	mentally ill	3,619979403	1,662432822
vset3_2_a1	A person gets flashbacks of their own car accident when watching a movie that also shows car accidents	F40-F48	mentally ill	3,835051546	1,662580903
vset3_1_a12	A person who gets lost in their thoughts		mentally healthy	3,37755102	1,665386281
vset8_1_a5	A person has difficulty sensing temperatures or pressure on their skin	F10-F19	mentally ill	3,223404255	1,666586592
vset11_1_a17	A person who regularly has difficulty falling asleep	F30-F39	ambiguous	3,716786818	1,669346552

vset4_2_a19	A person is quickly stressed by too many external stimuli, such as screaming children, and for this reason avoids using public transportation.	F50-F59	mentally ill	3,946236559	1,670495835
vset9_2_a17	A person makes sure several times that he or she has inserted his or her front door key after leaving his or her home	F40-F48	ambiguous	3,207920792	1,675212414
vset1_1_a5	A person is overly conscientious in all areas of life	F60-F69	ambiguous	3,400990099	1,675874765
vset6_1_a3	A person has problems with learning new things	F00-F09	ambiguous	3,075268817	1,676153458
vset5_2_a8	A person shows callous unconcern for the feelings of others	F60-F69	mentally ill	3,88	1,677419856
vset3_2_a18	One person has slept enough, but complains of fatigue the next day because the sleep was not restful	F50-F59	ambiguous	3,489583333	1,679566308
vset6_1_a11	A person new to a group wonders if they will be liked by the others		mentally healthy	3,074468085	1,686373992
vset8_1_a10	A person is strongly preoccupied with impressing others and attracting admiration for themselves	F60-F69	ambiguous	3,585106383	1,687458957
vset6_2_a5	A person the person is strongly irritable and anxious	F20-F29	ambiguous	3,893617021	1,687831752
vset3_2_a2	A person who has trouble falling asleep and staying asleep even though he or she is dull and tired	F30-F39	ambiguous	3,927835052	1,69095278
vset11_1_a12	A person thinks about their future with worry because it is uncertain		mentally healthy	3,15653965	1,69420053
vset6_2_a4	A person appears cold and distant to an extreme degree	F20-F29	mentally ill	3,35106383	1,695708986

vset10_2_a3	A person avoids interactions with other people because they feel they do not belong	F20-F29	ambiguous	3,867924528	1,696357283
vset4_2_a2	A person is afraid of blushing when meeting other people	F40-48	ambiguous	3,204301075	1,703954096
vset7_1_a8	A person has few social contacts and tends to withdraw	F20-F29	ambiguous	3,989361702	1,707004424
vset4_1_a8	A person is excessively resentful after offenses	F60-F69	ambiguous	3,279569892	1,709090728
vset2_1_a17	A person has been waking up several times during the night for months and has problems falling asleep again after these waking phases	F50-F59	mentally ill	3,885416667	1,709846356
vset7_1_a13	A person who hides in his/her bed in bad weather and rain		mentally healthy	3,20212766	1,713826049
vset5_1_a5	A person is euphoric and smiles throughout	F20-F29	ambiguous	3,42	1,718467722
vset5_2_a7	A person, although having slept sufficiently during the night, is so severely tired during the day that they need to sleep	F50-F59	mentally ill	3,81	1,721492188
vset4_2_a8	A person harbors deep distrust of his or her fellow human beings	F60-F69	mentally ill	3,838709677	1,727591453
vset4_2_a7	A person is excessively suspicious of other people	F20-F29	ambiguous	3,376344086	1,731510891
vset5_2_a9	A person who behaves self-centeredly and inconsiderately	F60-F69	ambiguous	3,5	1,732050808
vset6_2_a9	A person has trouble paying attention and remembering things	F10-F19	ambiguous	3,712765957	1,732810139
vset8_1_a4	A person is very susceptible to stress	F00-F09	ambiguous	3,712765957	1,732810139
vset9_2_a4	A person returns after leaving the apartment and checks if he/she has turned off the stove	F40-F48	ambiguous	3,188118812	1,736161693
vset3_1_a11	A person who is afraid of an important presentation		mentally healthy	3,255102041	1,736872557

vset3_1_a16	A person always goes to the toilet before leaving the home, even if he or she does not acutely feel the need to urinate	F40-F48	ambiguous	3,255102041	1,736872557
vset8_2_a15	A person sees two people laughing and looking in their direction and thinks they are making fun of them		mentally healthy	3,436170213	1,738609694
vset11_1_a10	A person does not feel like working and therefore calls in sick		mentally healthy	3,017525773	1,739690868
vset10_1_a5	A person generally relies on other people for life decisions and cannot make decisions alone	F60-F69	ambiguous	3,962616822	1,742505427
vset9_1_a16	One person looks at the clock, but forgets the time right away, so he or she has to look again		mentally healthy	3,441176471	1,743863523
vset6_2_a8	A person only makes contact with others where they know they are liked	F60-F69	ambiguous	3,212765957	1,746716369
vset6_1_a8	A person shows reticence in intimate relationships for fear of being shamed or ridiculed	F60-F69	ambiguous	3,872340426	1,74894157
vset3_1_a1	A person cannot concentrate properly for a few days after a car accident and feels disoriented	F40-F48	mentally ill	3,591836735	1,751798691
vset1_2_a17	A person avoids all eye contact with people he or she does not know	F40-F48	ambiguous	3,925373134	1,751971171
vset7_2_a1	A person who sleepwalks and can't remember it the next day	F50-F63	ambiguous	3,107526882	1,753644439
vset4_1_a17	A person has a reversed sleep rhythm so that they are awake and productive at night and asleep during the day	F50-F59	ambiguous	3,344086022	1,753844372
vset5_1_a15	A person buys an item of clothing that is actually outside their budget		mentally healthy	3,12	1,753956566

vset11_1_a15	A person is often so stressed that they forget to eat	F50-F59	ambiguous	3,702368692	1,754812103
vset7_1_a10	A person has a tendency to blame others	F60-F69	ambiguous	3,393617021	1,754981145
vset3_2_a8	A person to whom externals of people appear changed in shape or size	F20-F29	mentally ill	3,886597938	1,755208254

mentally ill

**item**	**statement**	**ICD-10-GM**	**a priori classification**	**mean**	**sd**
vset4_2_a6	A person feels that he or she cannot cope with the demands of daily life	F60-F69	ambiguous	4,086021505	1,564941407
vset6_2_a10	A person twists experiences by misinterpreting actions of others as hostile or contemptuous	F60-F69	ambiguous	4,574468085	1,569119757
vset4_1_a6	A person has a constant feeling of tension	F60-F69	ambiguous	4,193548387	1,596586471
vset5_1_a7	A person expresses very limited feelings and experiences little joy	F60-F69	mentally ill	4,92	1,606049171
vset5_1_a6	A person has a more depressed mood than average and life-weary thoughts come to mind	F30-F39	mentally ill	5,72	1,608562945
vset3_1_a5	A person who finds it very difficult to feel pleasure for themselves or others	F30-F39	mentally ill	4,214285714	1,613410296
vset7_2_a10	A person has a tendency to constant controls	F60-F69	ambiguous	4,79787234	1,61697838
vset5_1_a4	A person appears completely apathetic in social encounters	F00-F09	mentally ill	4,58	1,621696331
vset5_2_a2	A person often has an unfounded fear that someone close to them may die	F40-F49	mentally ill	4,74	1,624434634
vset9_2_a3	A person perceives his or her environment as unreal and feels disconnected from it	F40-F48	mentally ill	4,821782178	1,627243311

vset9_2_a9	A person has a constant feeling of apprehension	F60-F69	mentally ill	4,18	1,629153588
vset5_2_a5	A person has strongly erratic thoughts	F10-F19	ambiguous	4,08	1,631012575
vset5_1_a11	A person has the feeling of being under constant observation and control	F20-F29	mentally ill	5,11	1,675099876
vset4_2_a1	A person avoids all social encounters because he or she is too afraid of the possible rejection of those around him or her	F40-48	mentally ill	5,053763441	1,676990002
vset6_2_a1	A person repeatedly lapses into states of repeating the same, seemingly meaningless utterances for minutes after an unexpected bereavement in his or her immediate environment	F40-F48	mentally ill	4,819148936	1,678214399
vset10_2_a1	A person plays through situations in all conceivable facets in his or her head on a daily basis and is unable to detach himself or herself from them	F40-F49	mentally ill	4,682242991	1,685667276
vset6_2_a18	A person is so stressed and overwhelmed by their job that they get severe stomachaches and headaches when they leave for work in the morning	F50-F62	mentally ill	4,585106383	1,687458957
vset6_1_a7	A person injures himself or herself through cutting	F60-F69	mentally ill	6,032258065	1,690456672
vset6_2_a7	A person has mood changes that occur in short succession, changing from exuberant-joyful to depressed moods	F30-F39	mentally ill	5,063829787	1,696349722
vset3_2_a19	A person has had great difficulty falling asleep for months and therefore lies awake in his or her bed until the morning	F50-F59	mentally ill	4,164948454	1,699757411

vset11_1_a6	A person who sometimes finds his or her familiar surroundings strange or threatening.	F20-F29	ambiguous	4,798146241	1,700950815
vset1_2_a4	A person has great difficulty falling asleep and staying asleep for months at a time	F50-F59	mentally ill	4,487562189	1,703259971
vset8_1_a3	A person has a constant feeling of shortness of breath and hyperventilates	F40-F48	mentally ill	4,606382979	1,705261214
vset11_1_a16	A person who cannot motivate himself or herself to fulfill his or her obligations	F30-F39	ambiguous	4,084623323	1,705321304
vset8_2_a3	A person cannot detach himself or herself from a thought, although he or she actively tries to do so	F40-F48	ambiguous	4,074468085	1,705395371
vset1_1_a8	A person has a low frustration tolerance and a low threshold for aggressive behavior	F60-F69	ambiguous	4,228855721	1,708029035
vset1_2_a9	A person has repeated, frequently changing physical complaints over a period of years, examinations by various doctors remain inconclusive	F40-F48	mentally ill	4,417910448	1,710110409
vset1_2_a3	A person has a constant feeling of inner emptiness	F60-F69	mentally ill	4,925373134	1,711549878
vset5_1_a3	A person overeats and drinks without restraint	F00-F09	ambiguous	4,59	1,712078305
vset6_1_a6	A person is tired of life	F30-F39	mentally ill	5,372340426	1,716360506
vset3_2_a3	A person cannot remember things they did the day before	F00-F09	mentally ill	4,268041237	1,717043631
vset9_2_a2	A person has fear of panic attacks in public	F40-F48	mentally ill	5,00990099	1,717527581
vset2_2_a3	A person cannot enter into an interpersonal relationship because of feelings of their own inadequacy	F60-F69	mentally ill	4,747368421	1,719527527

vset6_1_a1	A person constantly self-diagnoses diseases that were previously ruled out by the treating doctors.	F40-F48	mentally ill	5,223404255	1,723676087
vset9_1_a17	A person doubts his or her own abilities so much that he or she is inhibited in his or her job because of it		ambiguous	4,294117647	1,726830986
vset9_1_a7	A person is often confused	F20-F29	ambiguous	4,450980392	1,727055819
vset1_2_a19	A person has the impression that certain occurrences in everyday life are meant only for him or her	F20-F29	ambiguous	4,099502488	1,732065169
vset7_2_a2	A person has an excessive fear of dying, even though there is no reason known to him or her for this thought	F40-F48	mentally ill	4,612903226	1,732253233
vset11_1_a7	A person who does not value himself or herself much	F30-F39	ambiguous	4,458290422	1,732366465
vset11_1_a3	A person who has had no interest in his or her friends and hobbies for months.	F30-F39	mentally ill	4,529351184	1,737373741
vset11_1_a4	A person is so afraid of public places that he or she does not leave his or her home	F40-F48	mentally ill	5,64742268	1,740496399
vset2_2_a4	A person has feelings of helplessness and dependence	F60-F69	ambiguous	4,547368421	1,742878647
vset3_1_a9	A person thinks that his or her thoughts can be heard aloud	F20-F29	mentally ill	5,285714286	1,752759239
vset10_2_a18	A person has a profound pattern of negative attitudes	F60-F69	mentally ill	4,345794393	1,754255224
vset9_1_a18	A person who is constantly exhausted and only with great difficulty gets out of bed at all	F30-F39	mentally ill	4,578431373	1,754407601
vset4_1_a1	A person is afraid of any large gathering of people	F40-F45	mentally ill	4,860215054	1,76062858

vset1_2_a7	A person feels as if he or she is wrapped in absorbent cotton and has difficulty making contact with the outside world	F40-F48	mentally ill	4,925373134	1,766183169
vset7_2_a18	A person believes narratives to be true that have no connection to reality	F00-F09	mentally ill	4,117021277	1,771072107
vset4_1_a5	A person, though there is cause for sadness, is carelessly cheerful	F30-F39	ambiguous	4,172043011	1,773130711
vset8_2_a7	A person wants to know permanently what the partner is doing and where he or she is.	F60-F69	mentally ill	4,35106383	1,776231645
vset1_2_a6	A person who feels a strong desire to use addictive substances	F10-F19	ambiguous	4,85	1,778571501
vset11_1_a5	A person feels that their thoughts can be followed and read by others	F20-F29	mentally ill	5,406797116	1,779121508
vset1_1_a3	A person who is uncomfortable with others getting emotionally close to him or her and cannot allow this to happen.	F60-F69	ambiguous	4,326732673	1,782216803
vset7_1_a7	A person has constant and plaguing feelings of doubt and is prone to perfectionism that limits them in the way they live their life	F60-F69	mentally ill	4,457446809	1,794045669
vset7_2_a3	A person repeatedly lapses into states of physical immobility after an unexpected bereavement in their immediate environment	F40-F48	mentally ill	4,85106383	1,795798243
vset5_2_a4	A person takes refuge in their own ideas so excessively that it is difficult to communicate with them.	F30-F39	ambiguous	4,76	1,798540373
vset9_1_a9	A person is not able to take responsibility for their actions and transfers this to their fellow human beings	F60-F69	ambiguous	4,460784314	1,800283633

vset2_2_a2	A person who seems permanently indifferent and apathetic to their environment, but feels very tense inside	F30-F39	mentally ill	4,768421053	1,801019992
vset8_2_a4	A person sees things that are not real, but also knows that they are not real	F00-F09	mentally ill	4,925531915	1,803457621
vset1_1_a6	A person uses only a limited part of his or her actual vocabulary	F00-F09	ambiguous	4,074257426	1,803653201
vset5_2_a11	A person suspects conspiracies by all their fellow human beings directed against them	F20-F29	mentally ill	5,24	1,809737969
vset8_1_a11	A person whose mood is consistently depressed even when their life circumstances improve	F30-F39	mentally ill	5,021276596	1,813798506
vset1_1_a7	A person feels as if behind a thick pane of glass in relation to their environment	F40-F48	mentally ill	4,702970297	1,815053441
vset9_2_a6	A person sees objects or people that are not real	F10-F19	mentally ill	5,831683168	1,816971694
vset9_2_a1	A person feels they can get by on 2 hours of sleep a night for a long period of time	F30-F39	mentally ill	4,089108911	1,817135162
vset5_1_a2	A person is worried that they are no longer in control of their life and thinks they are going insane	F40-F49	mentally ill	4,97	1,817173963
vset2_1_a6	A person has an excessive fear of being separated or abandoned by people, who are important to them	F60-F69	ambiguous	4,625	1,819485179
vset9_1_a5	A person performs seemingly senseless movements for no apparent reason	F00-F09	ambiguous	4,401960784	1,819910801
vset7_1_a6	A person who looks at the world in an exaggerated melancholic way and paints everything black	F30-F39	mentally ill	4,170212766	1,823610373

vset4_2_a5	A person who does not value themselves much and feels like a burden to their environment	F30-F39	mentally ill	4,774193548	1,824525145
vset3_1_a17	A person deliberately carries out a suicide in such a way that it can still be detected	F60-F69	mentally ill	5,397959184	1,826154745
vset1_2_a2	A person must incessantly repeat a word that is always the same in order to calm down	F20-F29	mentally ill	4,915422886	1,82970242
vset8_2_a1	A person who often feels guilty, even if they and their actions are unrelated to it	F30-F39	ambiguous	4,212765957	1,836735887
vset8_2_a2	A person is afraid to ride the bus alone	F40-F48	mentally ill	4,606382979	1,838758845
vset7_2_a5	A person is delusionally convinced of something that is not consistent with objective reality	F20-F29	mentally ill	4,85106383	1,84307741
vset10_1_a8	A person is strongly convinced of something that is not consistent with objective reality	F20-F29	ambiguous	4,457943925	1,849428131
vset8_2_a8	A person steals things that are not for personal use or enrichment	F60-F69	mentally ill	4,79787234	1,852518119
vset2_2_a7	A person cannot distinguish whether things are real or happening in his or her mind	F20-F29	mentally ill	5,715789474	1,854627094
vset8_1_a1	A person has a recurring dream that causes them anxiety	F50-F66	ambiguous	4,095744681	1,861142838
vset1_2_a5	A person repeatedly goes into debt for unnecessary purchases	F30-39	mentally ill	4,004975124	1,861444365
