# Supplementary material for: ‘Concept creep’ in perceptions of mental illness — an experimental examination of prevalence-induced concept change
Source: Eur Arch Psychiatry Clin Neurosci. 2024 Jan 17;276(1):33–7. doi: 10.1007/s00406-023-01737-0 (PMC12904865; doi:10.1007/s00406-023-01737-0)
Supplement: Supplementary file 2 — Supplementary file2 (DOCX 50 KB) [file 406_2023_1737_MOESM2_ESM.docx]

**Supplement**

Detailed Method Description

Population survey

To capture the concept of “mental illness”, a representative population sample (n=1031) rated short statements about different conditions and behaviours of people, on a 7-point Likert scale, indicating to what extent each statement represents mental illness. For this purpose, we initially developed 368 statements, which varied in intensity. We divided the statements a priori into three equal categories: mentally ill, ambiguous, and mentally healthy, one-third each. Statements from the mentally ill category were developed along the 5th chapter (mental and behavioural disorders) of the ICD-10-GM (e.g., “A person cannot distinguish whether things are real or happening in his or her mind.”). We excluded eating disorders (F50), sexual dysfunction (F52), postpartum mental or behavioural disorders (F53), intelligence disorders (F70-F79), developmental disorders (F80-F89), and disorders affecting children and adolescents from our statements. We also developed statements from the ambiguous category along the ICD-10-GM, but in an attenuated way (e.g., “A person has few social contacts and tends to withdraw”). Statements from the category mentally healthy were freely chosen (e.g., “A person smacks while eating”).

The 368 statements were divided into 10 sets of 36 or 37 statements (variable set) each, of which one set was randomly selected per participant, and a fixed set of 17 statements (constant set), which was shown to all 1359 participants. The three categories (mentally ill, ambiguous, and mentally healthy) were distributed equally among the 36 or 37 statements of the 10 variable sets. In addition, we made sure that the different disease patterns from the ICD-10-GM were evenly distributed among the sets. The 36 or 37 statements in each of the variable sets were presented in random order. After half of the variable set (i.e., after 18 or 19 statements) and at the end of the variable set, we included a Turing test to ensure that the participants were reading carefully. Each participant was first given one of the 10 variable sets of 36 or 37 statements, and then given the 17 statements of the constant set. Therefore, each of the rating participants saw 53 respectively 54 statements. For each of the statements, participants were asked to answer the question, "Is this condition part of a mental illness?". The question was answered on a 7-point Likert scale from "definitely not" (1) to "definitely" (7). The average interview duration was 15 minutes. After data cleaning, 1031 participants remained.

Each of the statements from the variable set was rated by 103 participants. In accordance with Levari et al. (2018), the mean of each statement’s ratings represents the objective rating of mental illness in the following. The 17 statements of the constant set were rated by all 1031 participants. This allowed us to estimate the consistency of the ratings within the totality of our participants. Inter-rater reliability was very high (Cronbach's alpha = 0.91, 95% CI [0.90, 0.91]). We used each statement's objective rating of mental illness to classify it into one of three categories. Statements whose objective rating of mental illness was greater than 4 and less than or equal to 7 were considered as mentally ill; statements whose objective rating of mental illness was greater than 3 and less than or equal to 4 were considered ambiguous; and statements whose objective rating of mental illness was less than or equal to 3 and greater than or equal to 1 were considered mentally healthy.

Then, from each of the three categories, those statements were selected whose objective rating of mental illness had the lowest standard deviation. Since we wanted to decrease the signal prevalence of the mentally ill statements in the experimental part of our study and therefore needed more healthy statements, we selected more healthy than ill statements. This resulted in the following distribution, following Levari et al. [1]: 113 mentally healthy, 80 ambiguous, and 80 mentally ill statements. These 273 statements were used as materials in the second part of the Study. The translated list of the 273 statements together with their mean and standard deviation is available within the Open Science Framework (osf.io/w7muh/).

Experiment

Between October and December 2021, 150 students took part in an experiment using these statements. We excluded people studying medicine or psychology to avoid professional bias. All participants provided written informed consent and received an incentive of 20 Euros. The study was approved by the ethics committee of Leipzig University (384/21-ek) and conducted in the rooms of the Clinic and Polyclinic for Psychiatry and Psychotherapy Leipzig under appropriate Corona protective measures. Two participants at a time were brought by a research assistant to a room equipped with two laptops. The research assistant was also present in the room during the study. We then gave participants an information sheet with instructions on the procedure. We explained to them that in the following they would be given various statements about different conditions or actions, on the basis of which they were to decide whether what was described as part of a mental illness or not. The participants could answer the question with "Yes" or "No"; corresponding keys were marked on the keyboard of the laptops.

For all participants, the experiment consisted of a total of 240 statements that they had to rate. On each trial, one statement appeared on the screen. Only after they answered the question "Is this condition part of a mental illness?" They saw the next statement. There was no time limit, but we had instructed the participants beforehand that they should rate as intuitively and quickly as possible. To maintain participants' attention span, we included a break after every 24 trials.

For the prevalence-induced part of the study, we divided participants systematically into two conditions: About half of the participants were assigned to the stable condition (n=71). In this condition, statements of all three categories (mentally healthy, ambiguous, mentally ill) occurred with equal probability across all 240 trials. With reference to Levari et al. [1], we refer to the probability of selecting a statement from the mentally ill category as the signal prevalence. Accordingly, in the stable condition, the signal prevalence was 33.3%. The other half of the participants (n=67) belonged to the decreasing condition. In this condition, the signal prevalence decreased steadily from the 4th set onward. That is, the signal prevalence was 33.3% on the trials 1-96; 25% on trials 97-120; 16% on trials 121-144; 8.3% on trials 145-168; and 4.12% in trials 169-240. Thus, toward the end, participants in the decreasing condition saw statements almost exclusively that belonged to the mentally healthy and ambiguous category according to the objective rating of mental illness.

After the participants had completed the experimental part of the study, the online survey tool RedCap was used to collect the socio-demographic data of the participants.

Statistics

We analyzed the data using a generalized linear mixed model using the R statistical software platform, version 4.0.2 (R Foundation for Statistical Computing). Corresponding to Levari et al. [1], we fit a binomial generalized linear mixed model to our data in R using the lme4 package [2]. The binary measurement of whether a statement was judged as “mentally ill” or “mentally healthy” served as the dependent variable. The two conditions, that we created (stable and decreasing conditions), served as independent between-participant variable. The trial number (1-240) and the objective norming measurement from the pre-study from “definitely not” (1) to “definitely” (7) served as independent within-participant variables. We used the Akaike information criterion (AIC) for choosing the best fit for the data. We captured conditional R2 for model performance [3]. We additionally calculated ORs with the sjPlot package, which provides a package to calculate OR for complex modelling [4].

**Supplementary Table 1**

*Socio-Demographic Characteristic of the Students (n = 138)*

| Sample | *n =* 138 |
| --- | --- |
| Gender |  |
| Men | 30 |
| Women | 106 |
| Divers | 2 |
| Age ^a^ |  |
| 18-24 | 92 |
| 25-39 | 46 |
| Study Subjekt |  |
| Humanities | 53 |
| Sport  Law, economics and social science  Mathematics, natural sciences  Health Sciences  Agricultural, forestry, and nutritional sciences,  Engineering sciences  Arts  Other study subjects | 0  52  14  4  1  1  2  11 |

^a^ We devided age into < 25 and >= 25.

**Supplementary Table 2**

*Odds Ratios of The Generalized Linear Mixed Model*

| Predictors | *Odds Ratios* | | *CI* | *p* |
| --- | --- | --- | --- | --- |
| (Intercept) | | 0.00 | 0.00-0.00 | <0.001 |
| Condition [Decreasing Prevalence Condition] | | 0.72 | 0.52 – 0.99 | 0.042 |
| Trial | | 0.12 | 0.06 – 0.23 | <0.001 |
| Norm_mean | | 194596.67 | 112669.54 - 336096.75 | <0.001 |
| Condition [Decreasing Condition] * trial | | 1.66 | 1.08 – 2.53 | 0.020 |
| Trial * Norm_mean | | 34.24 | 12.22 – 95.94 | <0.001 |
|  |  | |  |  |
| Random Effects |  | |  |  |
| σ^2^ | 3.29 | |  |  |
| τ_00_ _subject_nr_ | 0.77 | |  |  |
| τ_11_ _subject_nr.trial_ | 1.17 | |  |  |
| ρ_01_ _subject_nr_ | -0.04 | |  |  |
| ICC | 0.26 | |  |  |
| N _subject_nr_ | 138 | |  |  |
| Observations | 33115 | |  |  |
| MarginalR^2^ / Conditional R^2^ | 0.553 /  0.667 | |  |  |

*Note.* We calculated this table with sjPlot[4]

References

1. Levari DE, Gilbert DT, Wilson TD, Sievers B, Amodio DM, Wheatley T (2018) Prevalence-induced concept change in human judgment. Science (New York, N.Y.) 360(6396):1465–1467. https://doi.org/10.1126/science.aap8731

2. Bates D, Mächler M, Bolker B, Walker S (2015) Fitting Linear Mixed-Effects Models Using lme4. J. Stat. Soft. 67(1). https://doi.org/10.18637/jss.v067.i01

3. Nakagawa S, Schielzeth H (2013) A general and simple method for obtaining R2 from generalized linear mixed-effects models. Methods Ecol Evol 4(2):133–142. https://doi.org/10.1111/j.2041-210x.2012.00261.x

4. Daniel Lüdecke (2018) sjPlot - Data Visualization for Statistics in Social Science. Zenodo. https://doi.org/10.5281/zenodo.2400856
